# Supplementary material for: Long-Term Task- and Dopamine-Dependent Dynamics of Subthalamic Local Field Potentials in Parkinson’s Disease
Source: Brain Sci. 2016 Nov 29;6(4):57. doi: 10.3390/brainsci6040057 (PMC5187571; doi:10.3390/brainsci6040057)
Supplement: Supplementary file 1 [file brainsci-06-00057-s001.zip › brainsci-148446-supplementary/brainsci-148446-figures S1-S4.docx]

Supplementary Materials: Long-Term Task- and Dopamine-Dependent Dynamics of Subthalamic Local Field Potentials in Parkinson’s Disease

Sara J. Hanrahan, Joshua J. Nedrud, Bradley S. Davidson, Sierra Farris, Monique Giroux, Aaron Haug, Mohammad H. Mahoor, Anne K. Silverman, Jun Jason Zhang and Adam Olding Hebb


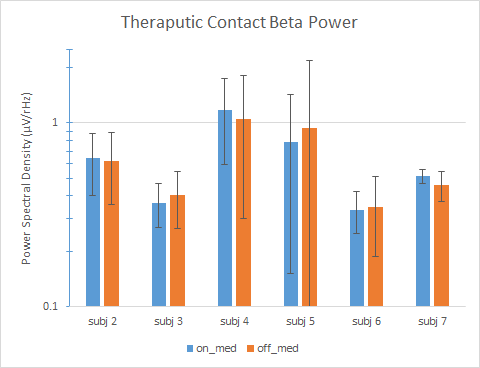


**Figure S1.** Beta frequency power of subthalamic nucleus (STN) local field potential (LFP) activity recorded with an implantable neurostimulator from therapeutic contacts with subjects in the medication on and off states at rest. Overall beta change was −0.28% ± 0.1% across all subjects. In three of six subjects, excluding subjects who exhibited an increase in beta frequency power, there was an average 9% ± 3% decrease in beta power in the medication on state. For all subjects, there was no significant difference between total beta power in the medication on state to the medication off state for the therapeutic contacts (paired student’s *t*-test). *p*-Values were 0.15, 0.18, 0.31, 0.31, 0.4, and 0.08 for Subjects 2, 3, 4, 5, 6, and 7, respectively. Subject 1 did not have a recording in the medication off state.


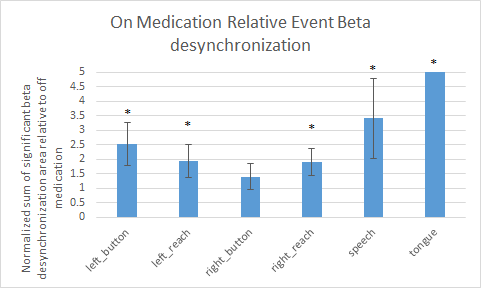


**Figure S2.** Event-related beta frequency desynchronization of subthalamic nucleus (STN) local field potential (LFP) signals in the medication on state relative to the medication off state. In four of five behaviours, there was a significant increase in the on medication sum of statistically significant beta desynchronization area relative to the medication off state with an event. Tongue values not shown: 39.75 ± 18.85.


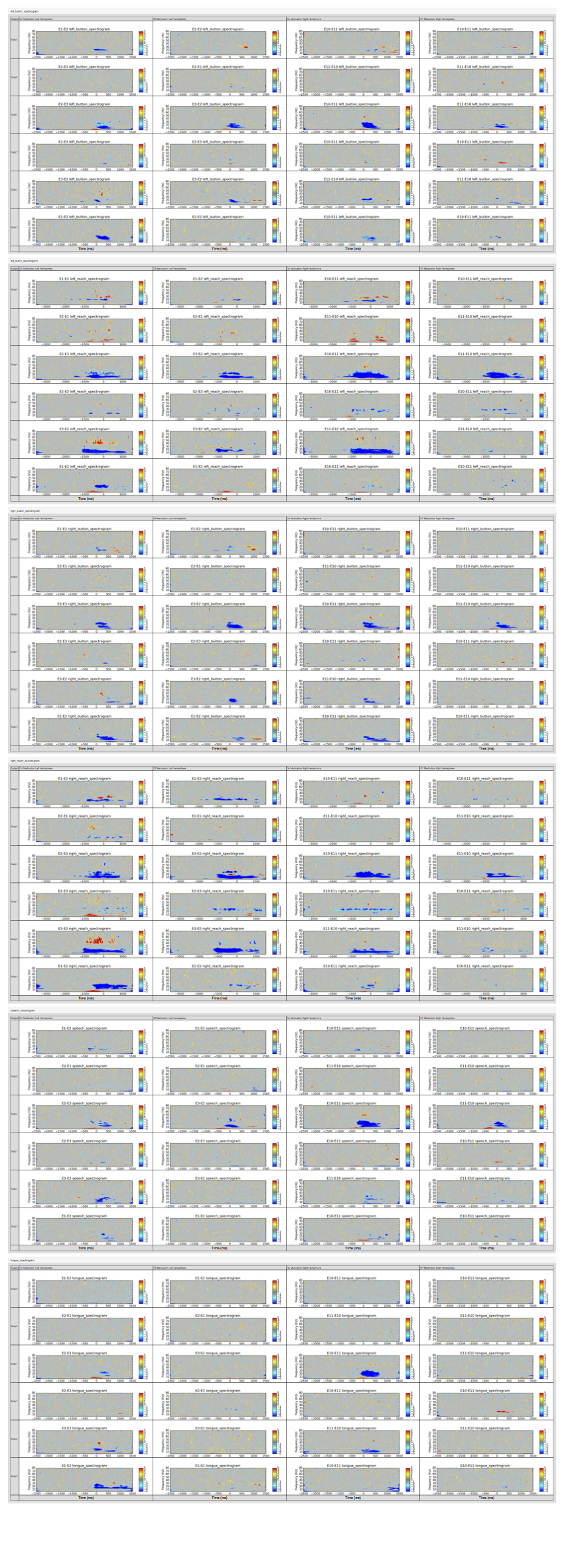


**Figure S3.** Dopamine dependent dynamics of subthalamic nucleus (STN) local field potential (LFP) activity recorded with an implantable neurostimulator (INS) with behavioural events. Subjects performed left and right button press, left and right reach, tongue extension, and speech. Medication on recordings were performed at three months post DBS lead implant. Medication off recordings were performed at six months post DBS lead implant. STN-LFP event related desynchronization (ERD) in beta (13–30 Hz) frequencies were observed in the medication off state. STN-LFP ERD in beta frequencies and event related synchronization in low gamma (35–70 Hz) frequencies were observed in the medication on state. One bipolar channel pair was selected from each hemisphere for behavioural recordings.


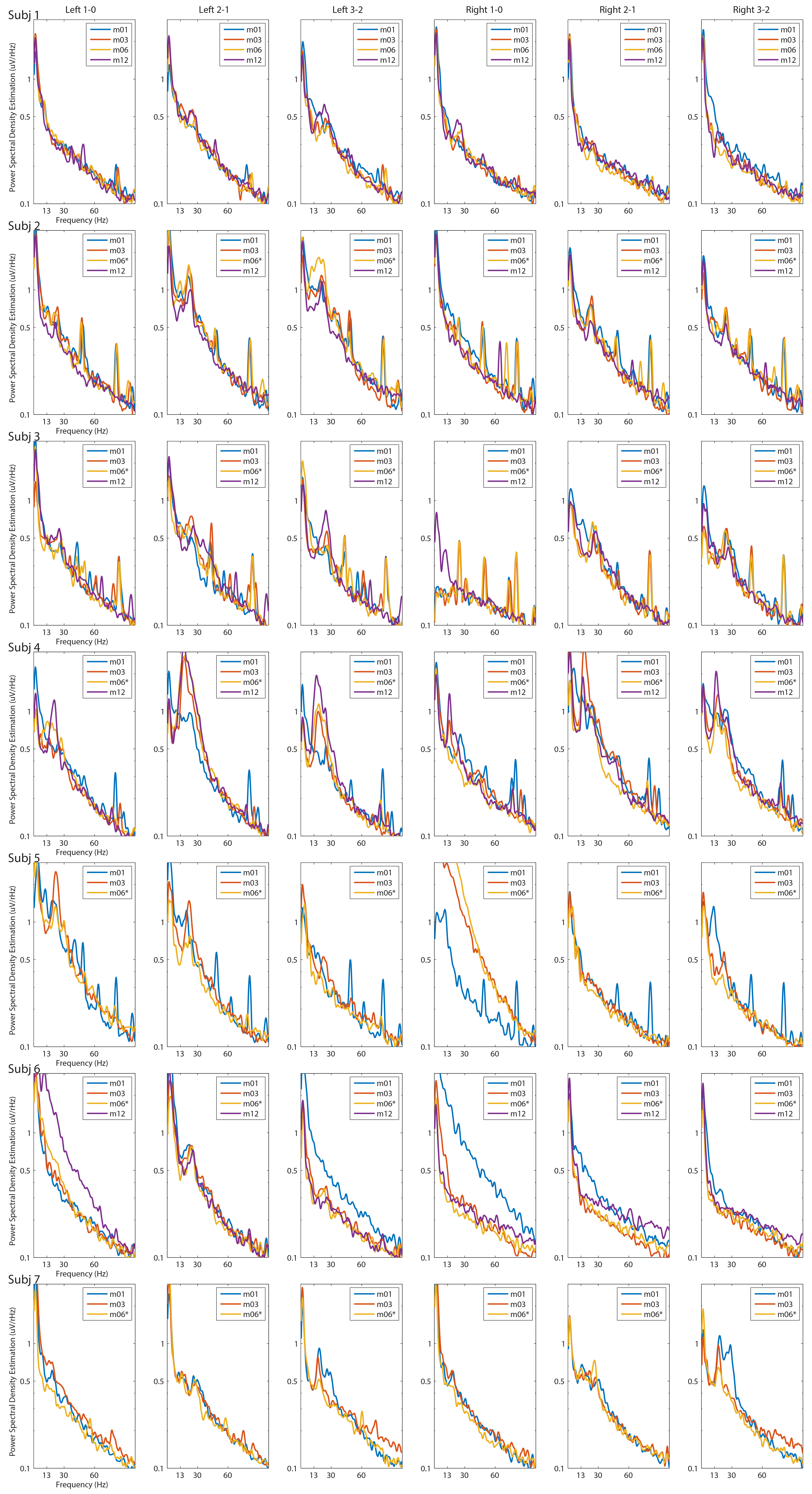


**Figure S4.** Stability of subthalamic nucleus (STN) local field potential (LFP) signals recorded with an implantable neurostimulator over one year. Representative PSD analysis of 30-s STN-LFP epochs with subjects at rest over one year from all bipolar pairs of electrodes are presented. STN-LFP frequency content was visibly consistent across multiple recording sessions. Legends denote recording date in months post-surgical implantation. * Denotes recording sessions in which the subject was in the medication off state.
